# Supplementary material for: Repeated evolution of self-compatibility for reproductive assurance
Source: Nat Commun. 2018 Apr 24;9:1639. doi: 10.1038/s41467-018-04054-6 (PMC5915400; doi:10.1038/s41467-018-04054-6)
Supplement: Supplementary file 1 — Supplementary Information [file 41467_2018_4054_MOESM1_ESM.pdf]

Supplementary Figures and Tables for "Repeated evolution of self-compatibility for reproductive assurance"

Nieuwenhuis, Tusso *et al.*

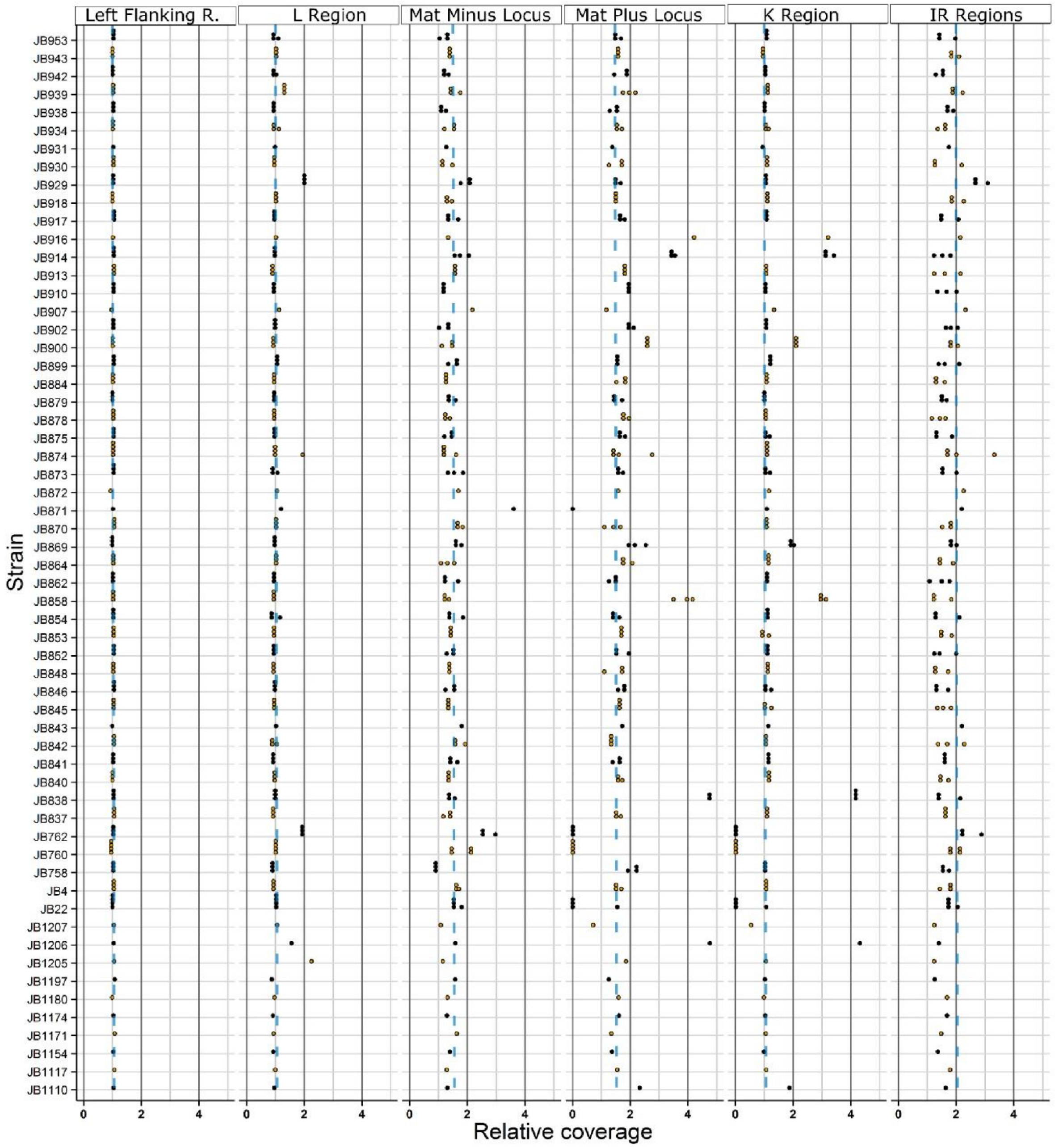

Supplementary Figure 1 | Relative coverage per base in each genome region/locus of the MTR. The mean coverage per region/locus was divided by the mean coverage of the flanking regions (see Supplementary Data 1 for raw coverage values and additional regions). Regions and loci are the same as in Fig 1a. Identical colors indicate replicates of the same strain. Expected relative coverage for standard h^{90} strain is shown as dashed blue lines.

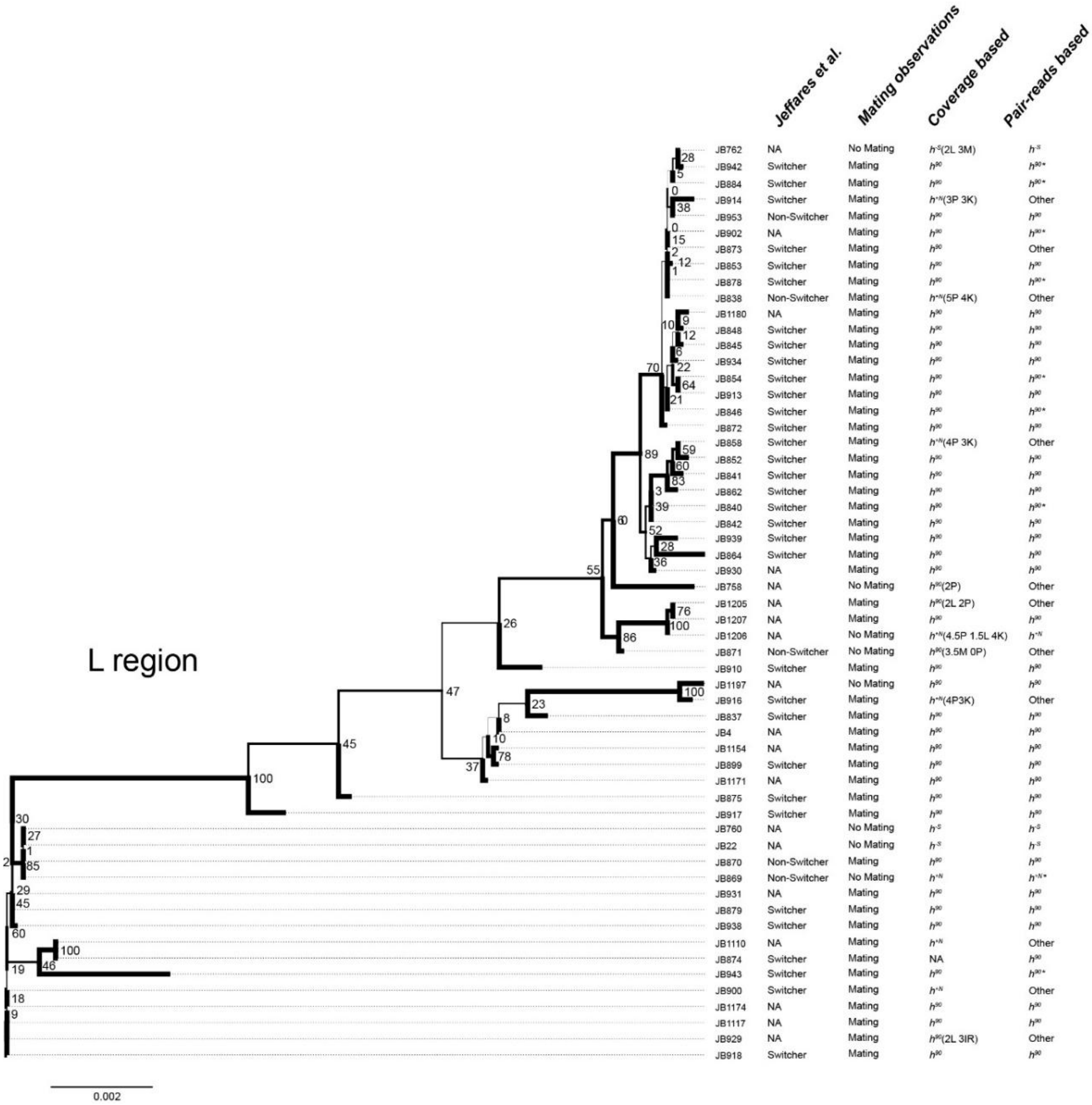

Supplementary Figure 2 | Unrooted maximum likelihood tree based on the L region with detailed information of phenotypes and strains. Extended version of Fig. 1b. Branch values show bootstrap support. Switching phenotype based on (i) Jeffares et al.[1] (ii) our observation of single-cell colony mating, and predicted based on genomic information from (iii) coverage and (iv) pair-read analyses. When genotypes differ in coverage from the expectations from standard configurations as given in Fig 1a, additional information is presented between brackets with predicted number of copies for differing regions/loci. Paired reads that fit to a standard configuration but without pair-reads for all the joint points of the configuration are indicated with an asterisk and those that do not follow a standard configuration are labelled ‘Other’. Scale bar units in substitutions per site.

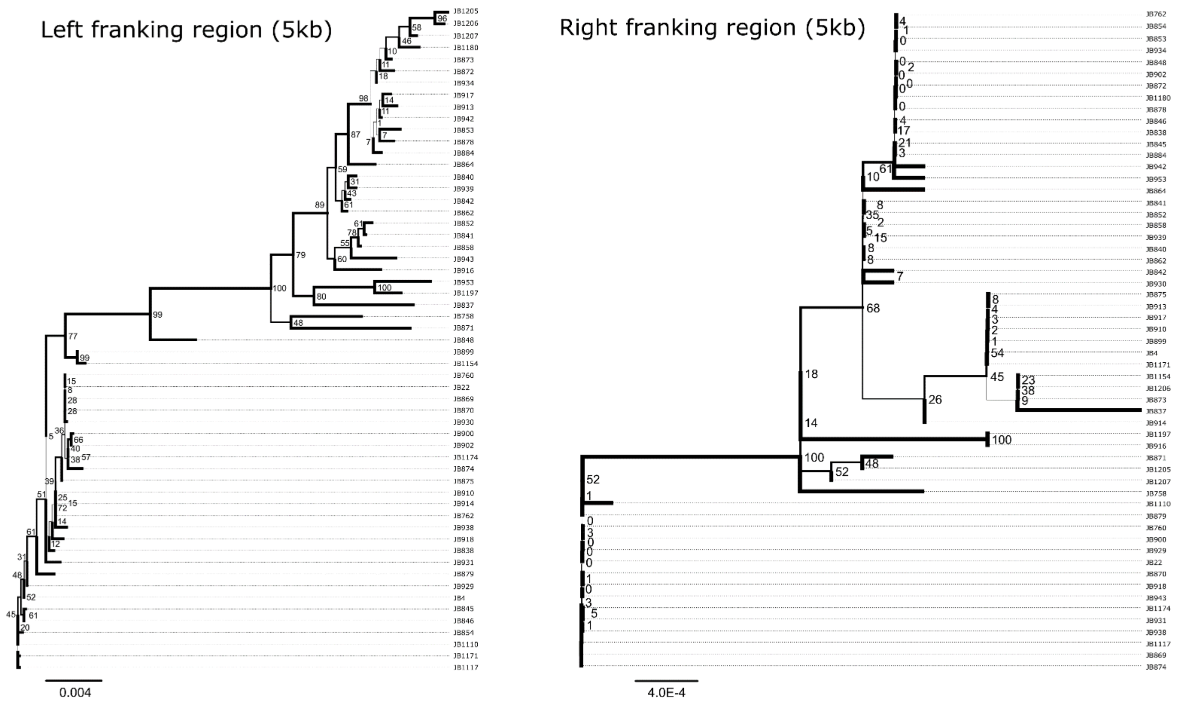

Supplementary Figure 3 | Unrooted maximum likelihood tree based on the left (centromere proximal of *mat1*) and right (centromere distal of IR-R) flanking regions of MTR. Branch values show bootstrap support. These trees also support two main clades as observed in analyses using the L region (Fig. 1b). Scale bar units in substitutions per site.

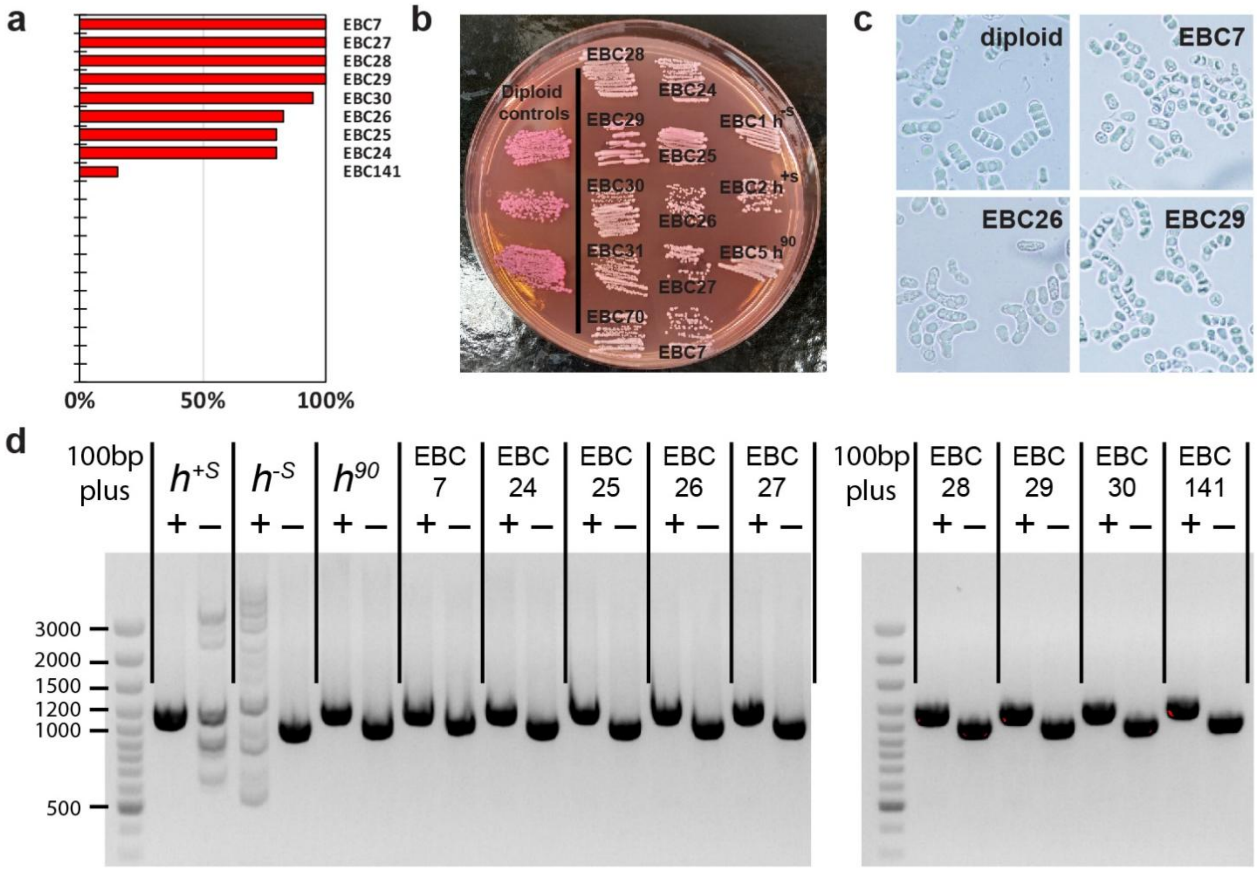

Supplementary Figure 4 | Sexual self-compatibility in evolved strains. (**a**) Percentage of switchers in 20 evolved populations after 25 generations. Values are calculated by counting the number of self-compatible single cell colonies using iodine vapour staining to mark mated cells in black from at least 100 individual colonies. Names on the right of the graph indicate the single cell cultures isolated from each population. (**b**) Evolved and control strains on YEA+Phloxin B staining diploid strains dark pink (controls in left most column). All evolved lines (two middle columns) show haploid phenotypes similar to haploid controls (right most column). (**c**) Ascus shape of diploid controls (top left panel) and three examples of evolved single-cell cultures. Irregular shaped asci indicate mating prior to meiosis and sporulation. (**d**) Amplification of the *mat1-P* and *mat1-M* mating-type regions for self-compatible single colony isolates of evolved strains. All evolved single cell isolates show both mating types at *mat1*, but the non-switching *P* and *M* controls (h^{+S} and h^{-S}) do not.

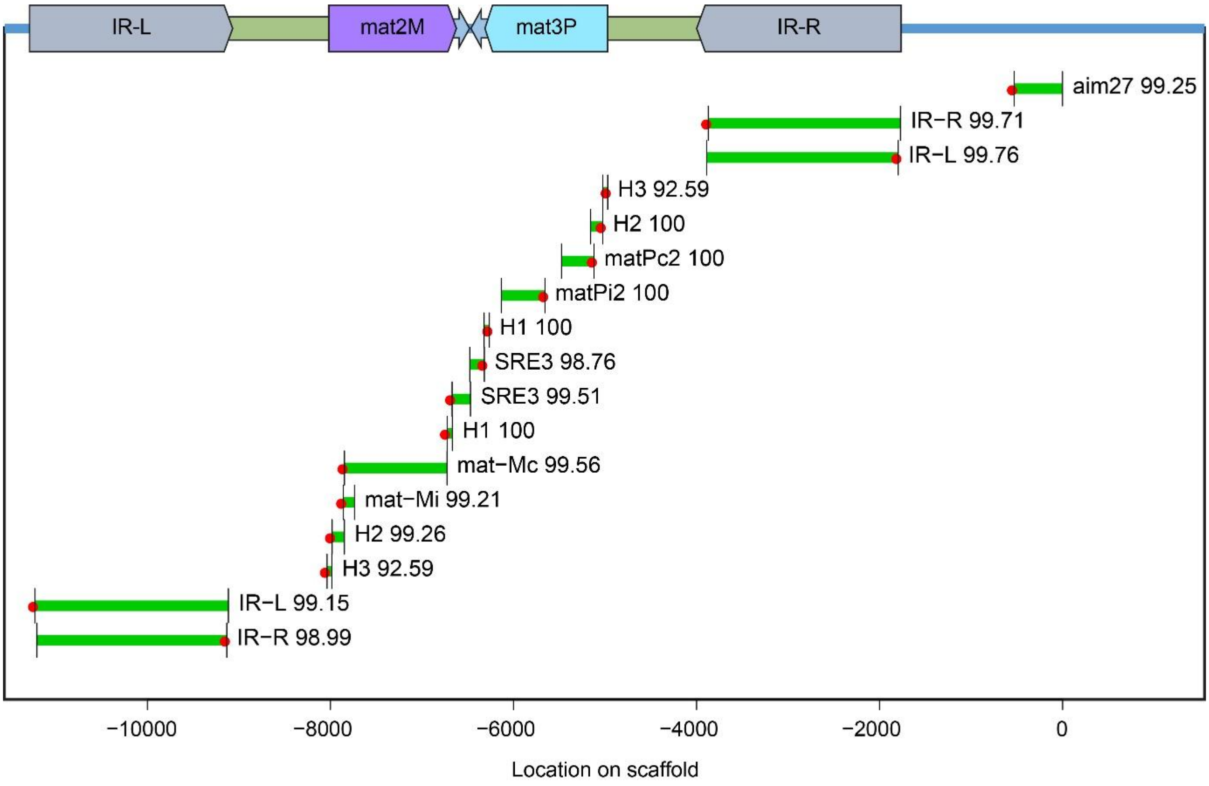

Supplementary Figure 5 | Overview of annotations within the silent mating-type region in the evolved strain EBC7. The location of each element is indicated by a green line with the red dot indicating the orientation relative to the reference genome[2] (ASM294v2; annotations and sequences derived from the MTR scaffold). The numbers after each element name indicate the sequence similarity with the reference genome. The location on the scaffold obtained from single molecule sequencing containing *mat1* (not in this image) and the novel silenced mating type region is given on the x-axis and is inverted to obtain standard orientation (centromere on the left). Note the inversion of the P cassette. The identity of the region between *IR-R* and *mat3P* is sequence identical to the reference region between *IR-L* and *mat2*.

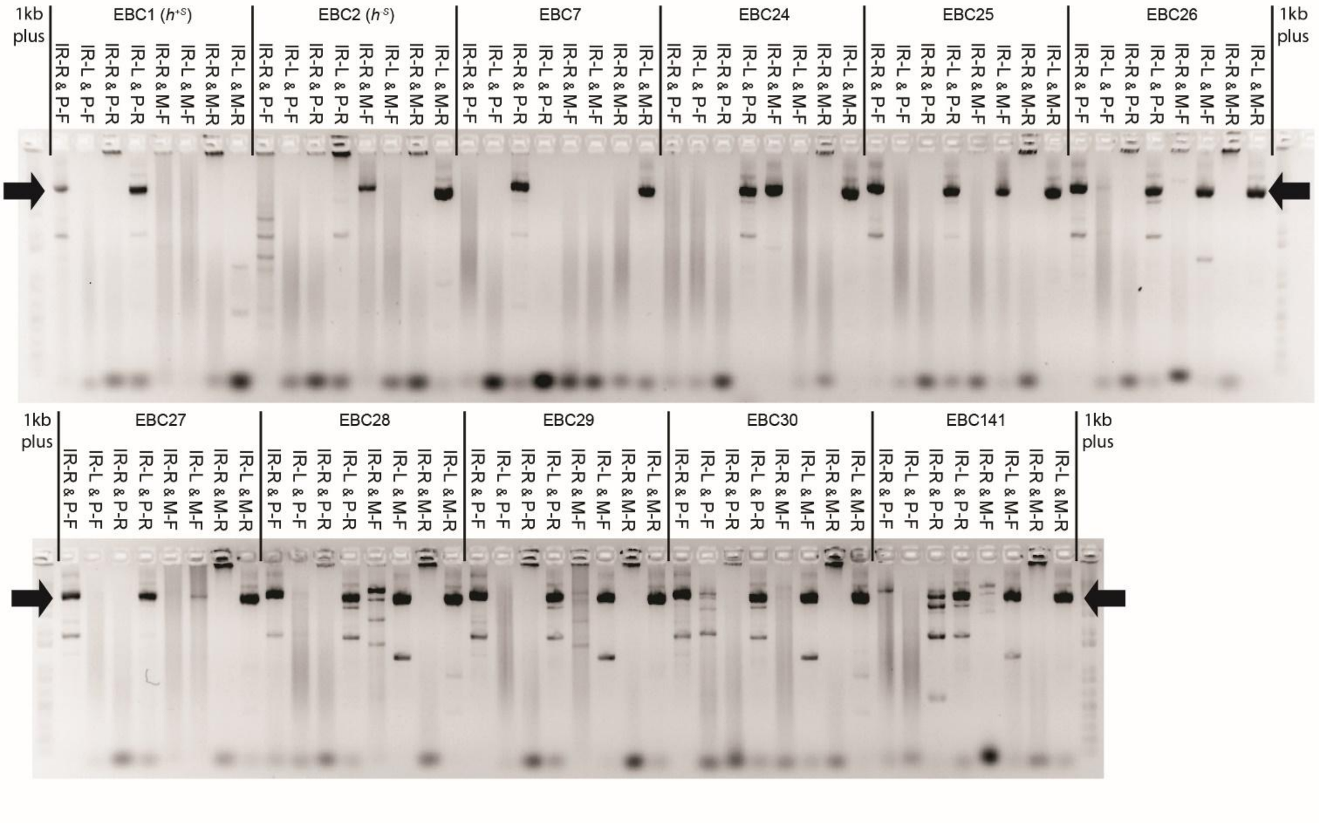

Supplementary Figure 6 | Regions flanking the silent mating-type show variation among replicate populations. Amplifications of the regions flanking the silent mating-type region (left of IR-L or right of IR-R) combined with M or P mating-type cassette specific reverse or forward primers (Mat-minus-F, Mat-minus-R, Mat-plus-F or Mat-plus-R). Strains EBC1 and EBC2 show only bands for either P or M in the expected direction. EBC7 is confirmed to have an M at IR-L and an inverted P at IR-R. The other strains all have more than two amplified fragments, which suggests that a duplication of the silent cassette occurred in different configurations, with EBC24 different from strains EBC25 to EBC141. Informative bands are at the height of the arrows. Other bands are non-specific amplicons due to complexity of the region. The variation among strains in presence of these bands suggests differences in mating type configurations between strains with similar target amplicons.

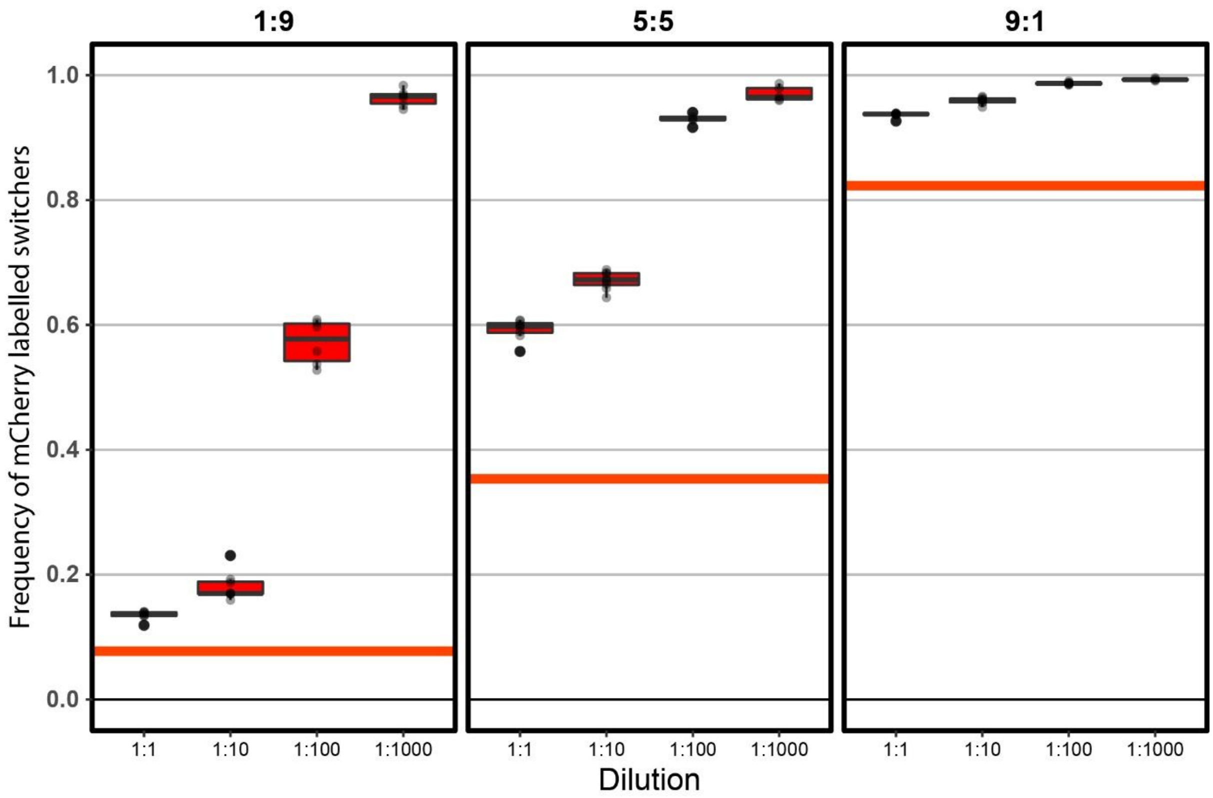

Supplementary Figure 7 | Frequencies before and after sexual competitions. The actual frequencies before competitions are indicated by the orange line. After mating, values are given by the red box. Gray light dots indicate individual measurements. Note that the frequency after competition at the highest dilution is always close to fixation.

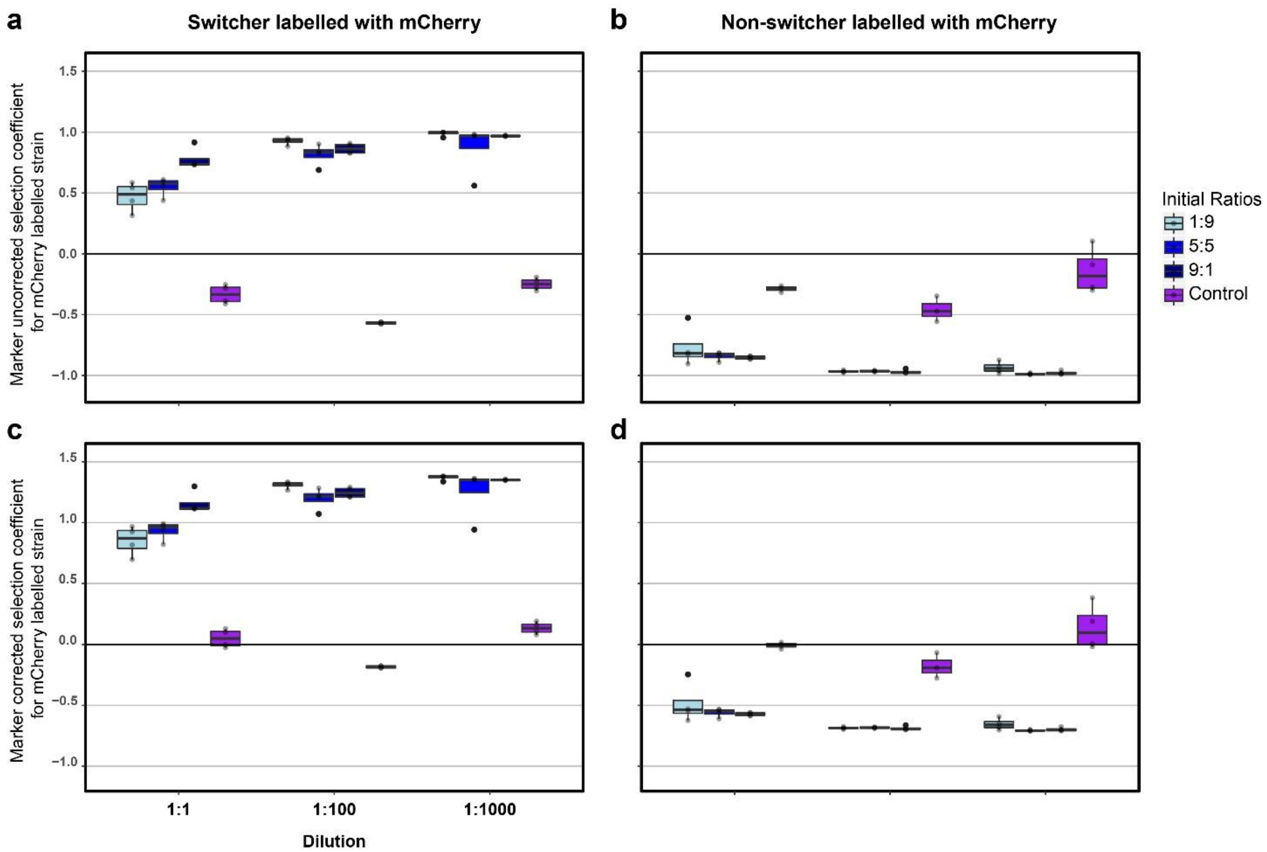

Supplementary Figure 8 | Fitness benefit of switchers during sexual competitions. Selection coefficients for mCherry labeled strains relative to unlabeled strains after one round of sexual competition. In each test, switchers (SW) and non-switchers (NS, P and M in equal ratios) are competed either with or without the mCherry-marker to account for marker effect (in the left panel the non-switcher is labelled with mCherry, in the right panels the switcher). The plots show that for each density and under all ratios the switchers increase relative to the non-switcher. Controls are between labeled and unlabeled isogenic strains. Each boxplot contains four biological replicates. The cell density in the undiluted sample was $3.2 \cdot 10^5$ cells mm^{-2}. Uncorrected for marker effect with (**a**) switcher mCherry marked or (**b**) non-switcher marked. Marker effect corrected with (**c**) switcher mCherry marked or (**d**) non-switcher marked.

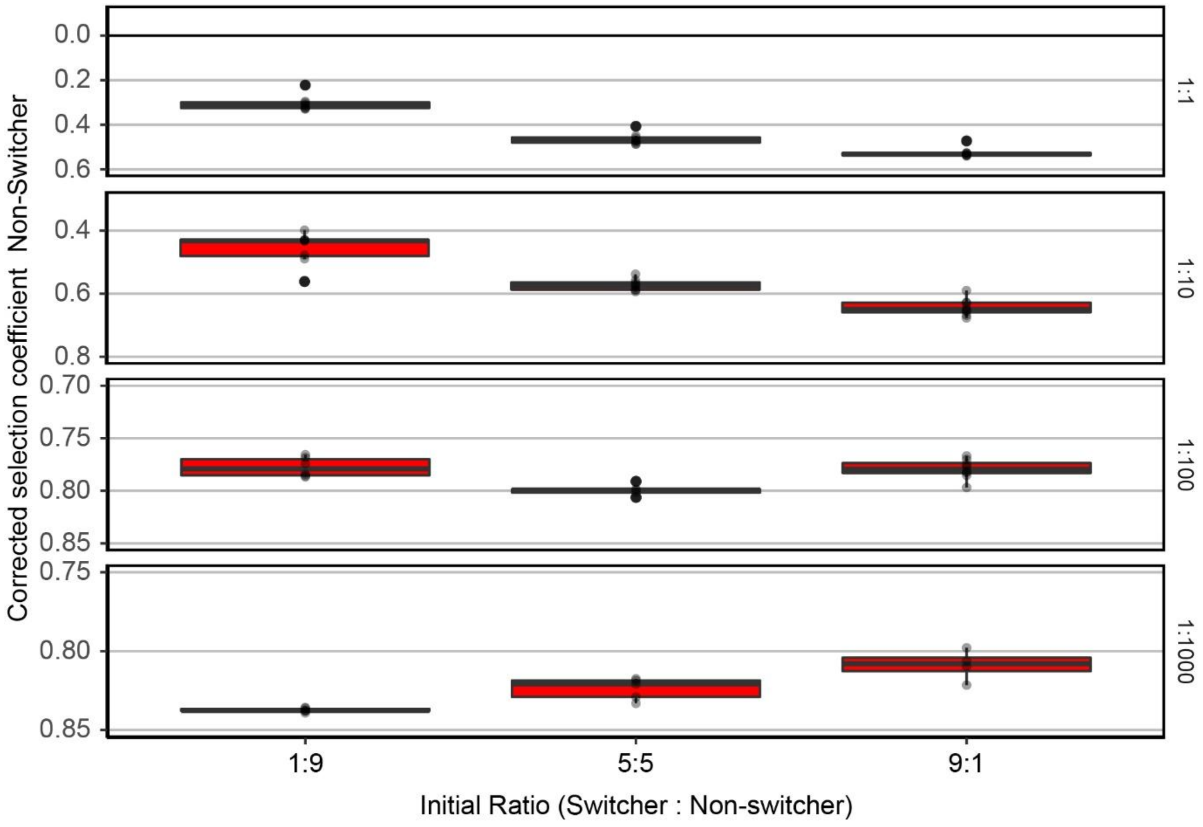

Supplementary Figure 9 | Effect of initial frequency on fitness cost to non-switchers during sexual competitions. Corrected selection coefficient of non-switcher labelled strains (inverted y-axis) after one round of sexual competition at three different initial frequencies under four dilution regimes shows that under high densities (undiluted and 1:10 dilution) mating-type switchers show positive frequency dependence, which under lower densities disappears and under the highest density appears to be reversed. Note the different scales on the y-axis between the panels.

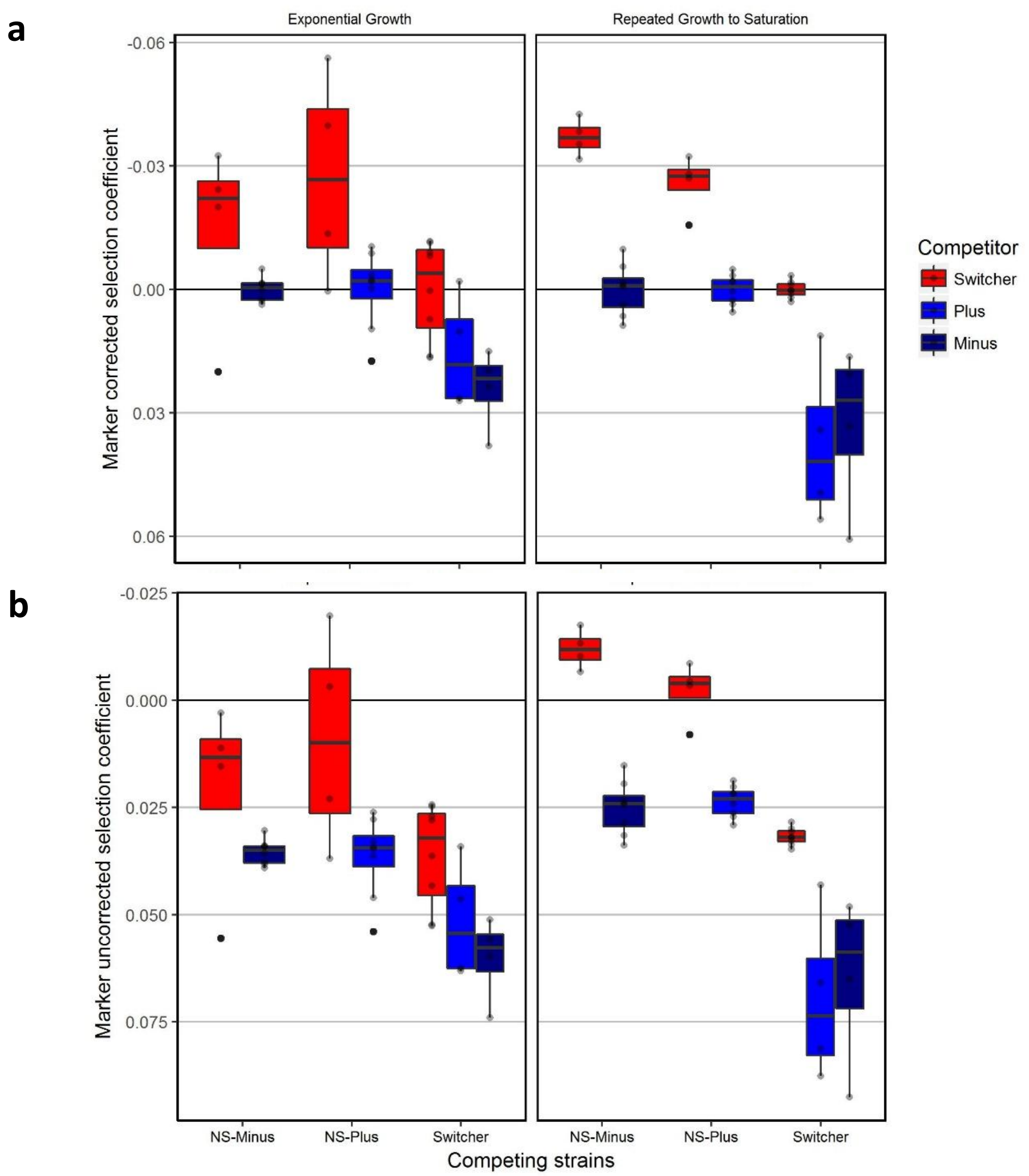

Supplementary Figure 10 | Fitness benefit of non-switchers during asexual competitions. Selection coefficients for the mCherry labeled strains relative to unlabeled strains under asexual growth, when maintained in exponential growth for five days (~45 generations; left panel) or after five transfers during which the population grew to saturation (~33 generations; right panel). In each test, switchers and the non-switching Plus or Minus strains are competed either with or without the mCherry-marker to account for the effect of the marker. Each boxplot contains four or eight data points. (**a**) Uncorrected for marker effect. (**b**) The data is corrected for marker effect. The assumption that the growth advantage of switching and the effect of the marker are additive is supported by the similarity in results, irrespective of the strains that is mCherry labelled.

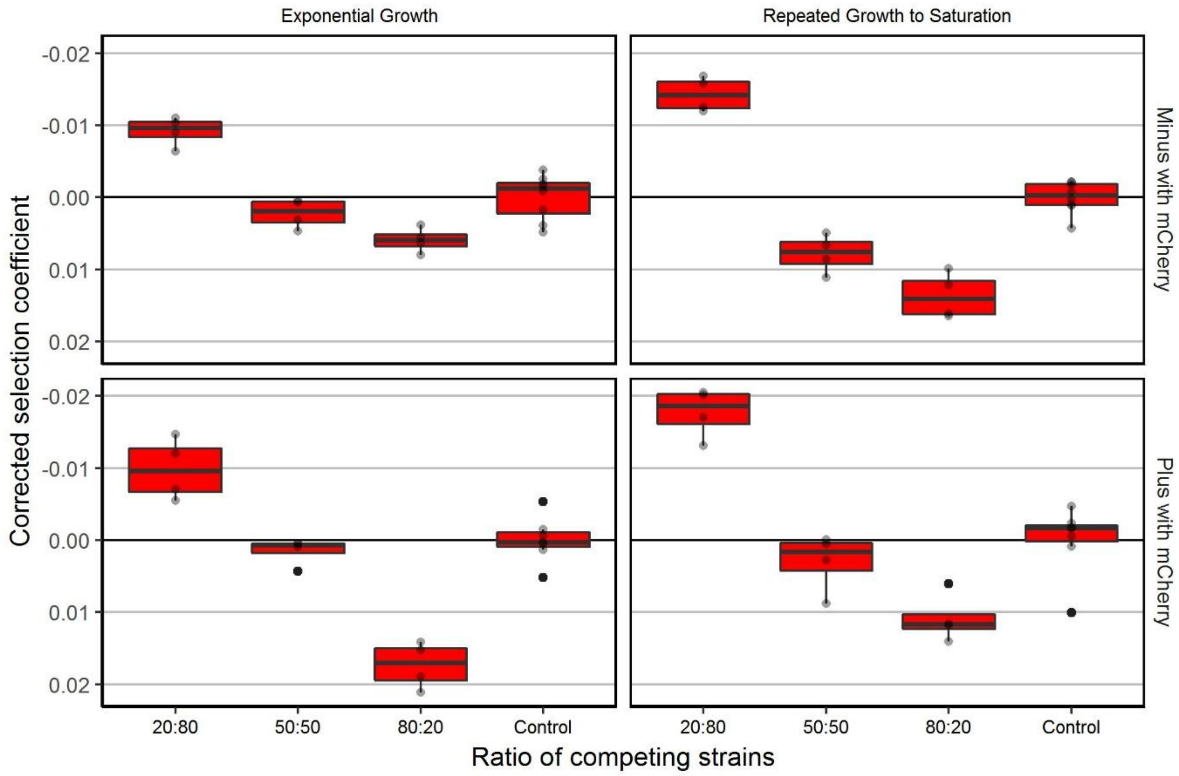

Supplementary Figure 11 | Frequency dependence in the asexual non-switcher strains. Competitions between Plus and Minus strains in different ratios (20:80, 50:50 and 80:20) show that the strain in the majority has an increased fitness relative to the 50:50 ratio, both in continuous exponential growth and repeated growth to saturation treatments. The values on the y-axis are the marker adjusted selection coefficients of the mCherry labelled strain relative to the unlabeled strain, on inverted y-axis to indicate increase or decrease in fitness. Top and bottom panels show either the Plus or Minus strain mCherry labelled, respectively. Controls are competitions between Plus-Plus and Minus-Minus in equal ratios, 4 replicates per boxplot.

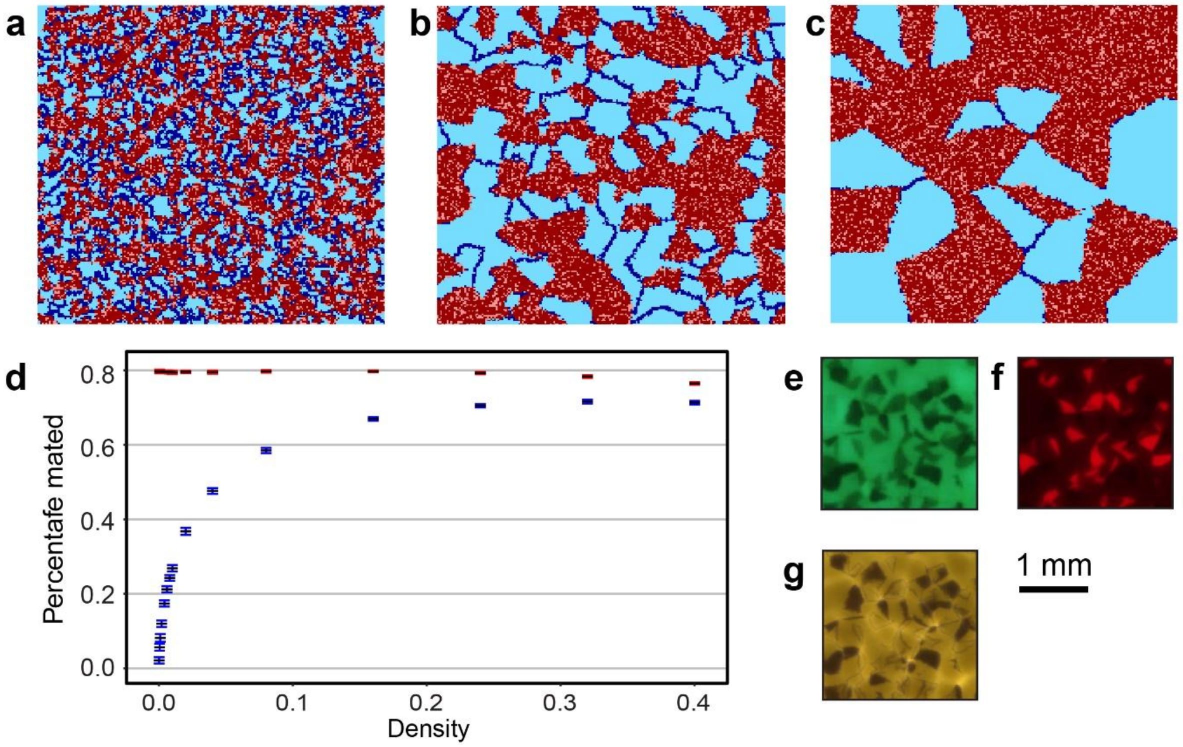

Supplementary Figure 12 | Effect of density on the mating potential of switching and non-switching strains. (**a**-**c**) Visualization of simulation showing the distribution of mated (dark) and unmated (light) cells in switching (red) and non-switching (blue) cells. Initial densities are 40% (**a**), 5% (**b**), and 0.5% (**c**) of area occupied. (**d**) Fraction of mated cells at different initial densities for switchers (in red always at top) and non-switchers (in blue always at bottom). Average (black lines) and standard deviation (whiskers) for 50 runs per density. (**e**-**g**) Low dilution mixture of switcher strain h^{90} and non-switchers h^{+S} mCherry and h^{-S} GFP, used in Fig 4. (**e**) Image in green channel. (**f**) Image in red channel. (**g**) Iodine stained plate, which showed mated cells (dark) and unmated cells (yellow). Staining was performed after fluorescence imaging, because iodine interfered with GFP fluorescence.

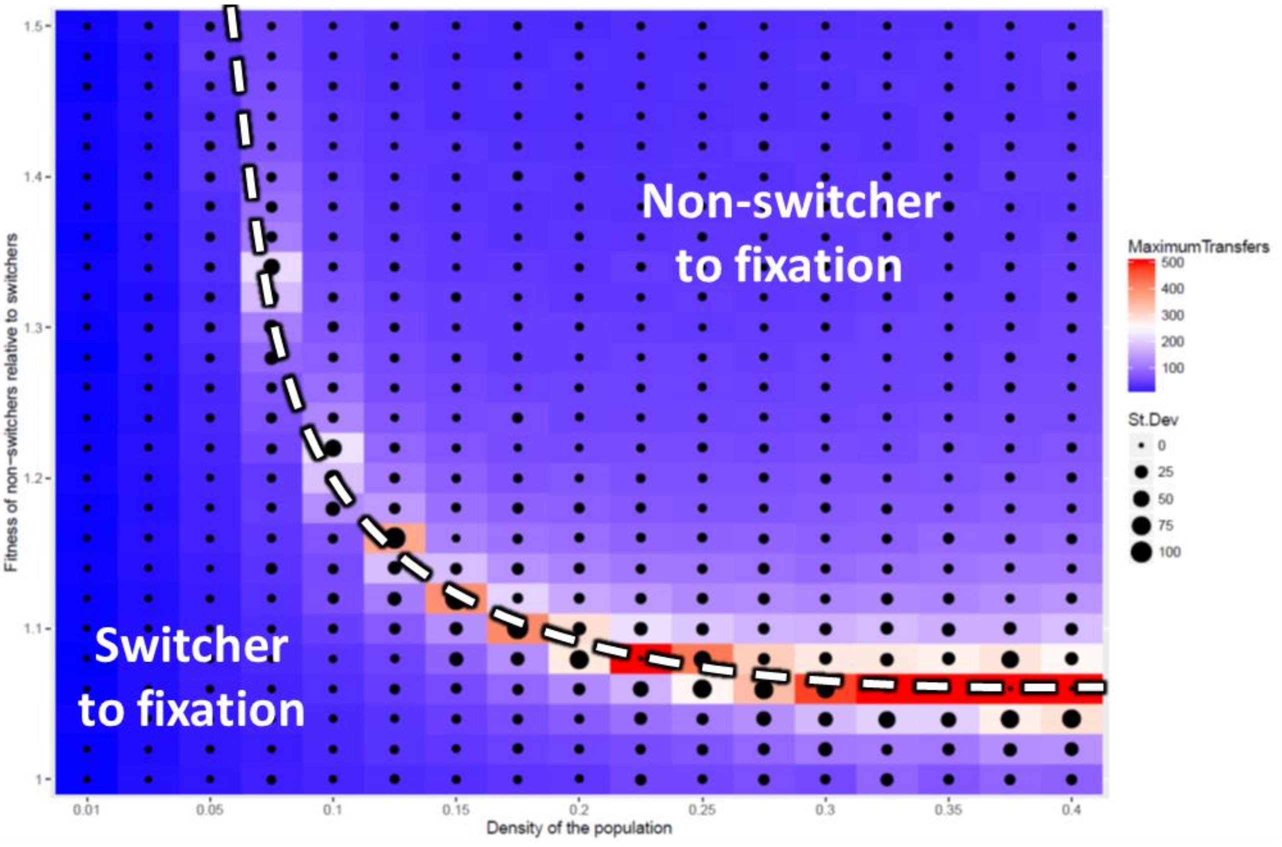

Supplementary Figure 13 | Effect of density and cost of switching on the speed of fixation of the switching on non-switching strategy. Heat map summarizing results from cellular automata simulations in a 40,000-cell grid after a maximum of 500 transfers with varying levels of population density and cost of switching. The color (see legend) indicates the number of generations required for one strategy to become fixed or remained polymorphic at 500 generations. The dashed line indicates above which values the non-switchers went to fixation (see Figure 4c). Size of the black dot indicates the Standard Deviation for ten runs per parameter combination.

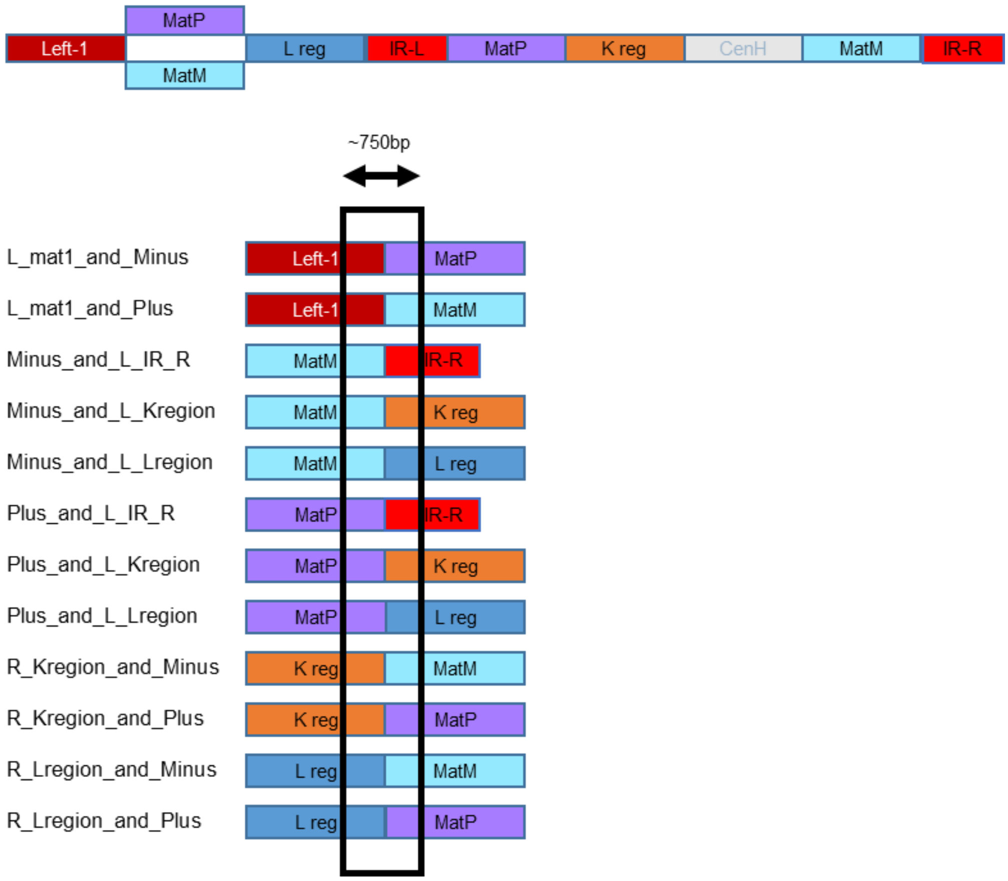

Supplementary Figure 14 | Schematic representation of the artificial scaffolds produced for paired-read inference of genotype. Figure at top gives an overview of the h^{90} configuration. The areas of the 12 combinations within the black box show the regions of the artificial scaffolds used for mapping (see Methods and Supplementary Data 1).

Supplementary Table 1 | Strains used in the experiments

Strain	Derived from	Mating-type region	Other
EBC1	EG441 [b]	h^{+S}	*ade6-M216*
EBC2	972h-	h^{-S}	*ade6-M210*
EBC3	972h-	h^{-S}	*ade6-M216*
EBC4	EG441	h^{+S}	*ade6-M210*
EBC5	968h90	h^{90}	*leu1-32*
EBC7	[c]	h^{EBC7}	*ade6-M216*
EBC24 to EBC30 and EBC141 [a]	[c]	$h^{EBC\#\#}$	*ade6-M210* or *ade6-M216*
EBC37 to EBC40	EBC5 [d]	h^{+N}	*leu1-32*
EBC41 to EBC44	EBC5 [d]	h^{-S}	*leu1-32*
EBC47	EBC5	h^{90}	*leu1-32 smm4R::kanMX6-P81nmt1-mcherry*
EBC59	EBC37	h^{+N}	*leu1-32 smm4R::kanMX6-P81nmt1-mcherry*
EBC60	EBC38	h^{+N}	*leu1-32 smm4R::kanMX6-P81nmt1-mcherry*
EBC61	EBC39	h^{+N}	*leu1-32 smm4R::kanMX6-P81nmt1-mcherry*
EBC62	EBC40	h^{+N}	*leu1-32 smm4R::kanMX6-P81nmt1-mcherry*
EBC63	EBC41	h^{-S}	*leu1-32 smm4R::kanMX6-P81nmt1-mcherry*
EBC64	EBC42	h^{-S}	*leu1-32 smm4R::kanMX6-P81nmt1-mcherry*
EBC65	EBC43	h^{-S}	*leu1-32 smm4R::kanMX6-P81nmt1-mcherry*
EBC66	EBC44	h^{-S}	*leu1-32 smm4R::kanMX6-P81nmt1-mcherry*

[a] Labelling of strains is non-consecutive due to isolation at different moments.

[b] Nielsen and Egel strain collection

[c] Single cell isolate from evolution experiment

[d] Spontaneous mutant for mating-type

Supplementary Table 2 | Primers used for PCR

name	Sequence (5` - 3`)	T_m (in °C)
mat1_fwd	5` - AGAAGAGAGAGTAGTTGAAG - 3`	52
mat-plus-F	5` - TGTGCGTATTATGGCTTGGTG - 3`	52
mat-plus-R	5` - CGGTAGTCATCGGTCTTCCA - 3`	52
mat-minus-F	5` - ACACCTACCTTGCACTCACA - 3`	52
mat-minus-R	5` - CCACATCTCTCCAACCAGCT - 3`	52
mat2:3-1-F	5` - AACATGTTCCTTCGCCTACG - 3`	59
mat2:3-1-R	5` - GACAGACTATCGGCCAAACAA - 3`	59
mat3:1-1-F	5` - TTAACAGGTGCTTGCTTGGC - 3`	59
mat3:1-1-R	5` - GTAGAAGGGCGCACACAAAA - 3`	59
mat1l-K-1-F	5` - TGGGATGAGTGCTTGCTTTG - 3`	59
mat1l-K-1-R	5` - TCGACGGATATGATCTACAATGC - 3`	59
mat3_very_L_R1	5` - GTTCTTCTTACCCGGATAGACA - 3`	60
mat3_very_L_R2	5` - GCAGCGTTGAGATAACTTGC - 3`	60
mat3_very_L_R3	5` - TGTCTATCCGGGTAAGAAGAAC - 3`	60
IR-L-left-Fwd	5` - ATCGCTTGTTTCCCATAGCA - 3`	60
IR-R-right-Rev	5` - CCGTTTCAGGTTCGGAGATA - 3`	60
Ssm4_right_Fwd	5` - AAGAGTCATCAGTGAGAGCCATCATTTCATAA GAGACCGCAGTATAAGAGTCGGGGCATGTAACAT TGGTAGCATAATCGCAACCAGAGCTAGAGCAGTT GAATTCGAG CTCGTTTAAAC - 3`	
Ssm4_right_Rev	5` -TTGCGAACTCAACCTACATCTCGATACTAGCGG TTTCCCCATCACTGTTGAAGGTGTTAATGGCACTG GCCGATTCATTCTCTTCAACCTCTCCTCCGTCTTG TACAGCTCGTCCATGCC - 3`	

Supplementary References

1. Jeffares, D. C. *et al.* The genomic and phenotypic diversity of *Schizosaccharomyces pombe*. *Nat. Genet.* **47,** 235–241 (2015).
2. Wood, V. *et al.* The genome sequence of *Schizosaccharomyces pombe*. *Nature* **415,** 871–880 (2002).
