# Supplementary material for: Repeated evolution of self-compatibility for reproductive assurance
Source: Nat Commun. 2018 Apr 24;9:1639. doi: 10.1038/s41467-018-04054-6 (PMC5915400; doi:10.1038/s41467-018-04054-6)
Supplement: Supplementary file 3 — Description of Additional Supplementary Files [file 41467_2018_4054_MOESM3_ESM.pdf]

Description of Additional Supplementary Files

File Name: Supplementary Data 1
Description: Raw data for genotype prediction. Mean coverage per base in each region or locus. The mean coverage in each region was divided by the mean of the flanking regions (left and right flanking region: base coverage) to obtain the relative coverage per region (values plotted in Supplementary Fig. 1). The number of raw pair read that supports different genetic configurations are shown in the second half of the table. It differentiates the number of pair reads using Bowtie and BWA. Genetic regions as in Fig. 1 and Supplementary Fig. 13. L_mat1_and_Minus: 3' flanking region linked to mat minus, L_mat1_and_Plus: 3' flanking region linked to mat plus, Minus_and_L_IR_R: mat minus linked to 3' side of IR-R, Minus_and_L_Kregion: mat minus linked to 3' side of K region, Minus_and_L_Lregion: mat minus linked to 3' side of L region, Plus_and_L_IR_R: mat plus linked to 3' side of IR-R, Plus_and_L_Kregion: mat plus linked to 3' side of K region, Plus_and_L_Lregion: mat plus linked to 3' side of L region, R_Kregion_and_Minus: 5' side of K region linked to mat minus, R_Kregion_and_Plus: 5' side of K region linked to mat plus, R_Lregion_and_Minus: 5' side of L region linked to mat minus, R_Lregion_and_Plus: 5' side of L region linked to mat plus.
